# Challenges to global measles eradication: is it all in the timing?

**DOI:** 10.11604/pamj.supp.2017.27.3.12553

**Published:** 2017-06-21

**Authors:** Robert Davis, William Baguma Mbabazi

**Affiliations:** 1 American Red Cross, PO Box 41275-00100, Nairobi, Kenya; 2 African Field Epidemiology Network, Lugogo House, Plot 42, Lugogo By-pass, P.O. Box 12874 Kampala, Uganda

**Keywords:** Global measles eradication, timing, Uganda

## Abstract

The case for global eradication of measles was first made in 1982. Since then, technical aspects of measles eradication have concluded that measles satisfied all criteria required for eradication. To date, only smallpox, among human diseases, has been eradicated, with polio, the next eradication candidate. In all previous eradication programmes, the pattern of slow implementation and missed deadlines is similar. Lessons from these past eradication programs should inform development of a time-limited measles eradication program. Notably, no measles eradication resolution is likely until member states are satisfied that polio eradication is accomplished. However, there is an impetus for measles eradication from the western hemisphere, where governments continue to pay the high costs of keeping their region measles free until global measles eradication is achieved. While previous vaccine preventable diseases eradications have depended on supplemental immunizations (SIAs), measles eradication will have to build both on SIAs and routine immunization systems strengthening. This article reviews non-technical considerations that could facilitate the delivery of a time-limited measles eradication initiative. The issues discussed are categorized as a) specificities of measles disease; b) specifics of measles vaccine/vaccination; c) special considerations for endemic countries and d) organization of international partnerships. The disease and vaccine specific issues are not insurmountable. The introduction of routine measles second dose, in the context of EPI systems strengthening, is paramount to endemic developing countries. In the international partnerships, it should be noted that i) Measles eradication will be easier and cheaper; ii) the return on investment is compelling; iii) leverage is feasible on the experiences of the Measles/Rubella initiative; iv) two disease eradication targets in one initiative are feasible and v) for the first time, an eradication investment case will inform the decisions. However, if previous eradication efforts have been marathons, measles eradication will need to be a sprint.

## Essay

Eradication, as distinguished from control and elimination, is defined as the reduction to zero of the incidence of a given disease and the elimination of the etiologic agent, so that fresh transmission is impossible. Elimination refers to the clearance of a disease from a defined geographical area, with the threat of reestablishment of endemic or epidemic transmission as a result of importations from areas with ongoing transmission or other sources [1]. To date, only smallpox, among human diseases, has been eradicated, with polio, the next eradication candidate, now endemic in only three countries. Table 1 shows the timeline for the three diseases targeted for global eradication by the World Health Assembly. The pattern of slow implementation and missed deadlines is dismayingly similar for all three [2]. This article will review facilitating factors and challenges to the kind of time limited international initiative which could bring measles transmission to an end.

**Table 1:** Vaccine preventable diseases targeted for global eradication by the WHA

	**First W.H.A. eradication resolution**	**Current status**
Malaria	1955	Not eradicated; WHA resolution of 1969 refers.
Smallpox	1959	Eradicated; last naturally occurring case, 1977
Polio	1988	Three countries remain endemic for wild poliovirus

Country level interruption of measles transmission was first achieved in the Republic of the Gambia in the 1960s [3], shortly after the 1963 licensing of the measles vaccine. It was in 1982 that Hopkins and colleagues made the case for global eradication of measles [4]. Subsequent decades have seen the passage, at the regional level, of measles elimination resolutions by each of the six W.H.O. regions. Only one of the six regions has eliminated local transmission of the measles virus. There is, as of this writing, no global (World Health Assembly) call for measles eradication. A 2011 call by former UN Secretary-General Kofi Annan for a global measles eradication goal [5] was not successful. No measles eradication resolution is likely until member states are satisfied that polio eradication is either accomplished or, at least, close to realization [6].

There is, however, an impetus for eradication from the western hemisphere, where governments will continue to pay the high costs of keeping their region measles free until global measles eradication is achieved. Writing in The Lancet Public Health, Durrheim and Crowcroft have set down the case for early measles eradication in terms of economies to all concerned, especially governments of the western hemisphere [7].

In their 2010 article, “Biological Feasibility of Measles Eradication” [8], Moss and Strebel reviewed the biological criteria for eradication, as follows: 1) Humans are the sole pathogen reservoir; 2) Accurate diagnostic tests exist; 3) An effective, practical intervention is available at reasonable cost.

The year 2010 also saw a three day meeting, in Washington, of an ad hoc advisory group on measles eradication, hosted by the Pan American Health Organization. The proceedings of that consultation [9], more detailed than those of Moss and Strebel, drew similar conclusions. Meeting in Geneva later in 2010, the Strategic Advisory Group of Experts (SAGE) reviewed the findings of the Washington consultation and endorsed its recommendations [10].

The purpose of the present article is not to review the technical aspects of measles eradication, which were reviewed thrice in one year, [8-10] and all three review authors concluded that measles satisfied all three technical criteria, and in their words, “the challenges for measles eradication will be logistical, political, and financial.” The present article will focus primarily on non-technical aspects of measles eradication.

## Issues in measles eradication

This discussion will cover three sets of issues pertinent to measles eradication namely: 1) Issues specific to the disease and the vaccine; 2) Issues specific to endemic countries; 3) Issues specific to international partners.

### Issues specific to the disease and the vaccine

Measles has several challenging characteristics which set it off from other infectious diseases. The basic reproductive Rate (R0) for measles is usually placed at 9 to 18, making it the most contagious of common diseases; Measles is transmissible during the prodromal period, so that undetected cases can spread the disease. The virus persists in the air for hours, so that health facility waiting rooms are a potential focus of transmission, even after the infected child has departed; Nosocomial transmission is well documented [11] and yet many countries do not require that health workers provide proof of measles immunity as a condition of employment.

Most hospitals do not routinely administer measles vaccine to unvaccinated children upon admission. The minimum level of vaccination coverage needed to assure herd immunity and interruption of transmission (89-94 percent) is correspondingly high, and 95 percent coverage is often used as an operational objective in “herd immunity” planning documents; The measles vaccine is safe and effective, but not fully protective when given at 9 months of age, as is the practice in most developing countries. Seroconversion is a function of age, and is appreciably higher at 12 months and in the second year of life. Stopping transmission means moving to a two-dose dose regime, preferably delivered in the first and second years of life; Children whose mothers were vaccinated against measles, rather than having been infected with wild measles virus, are liable to have a shorter period of passive protection from the disease during infancy since the quantity of antibodies transmitted across the placenta is lower than in persons who have had measles disease [12].

On the positive side, there are no known chronic measles virus excretors. Nor can the live vaccine virus survive in the environment or be transmitted from person to person. In addition, measles seroconversion is independent of the enteroviruses which sometimes hamper vaccine response with oral polio vaccine. Hence, three potential challenges commonly associated with polio eradication do not apply to measles.

While there are issues with vaccination in HIV-seropositive individuals, they do not preclude vaccination [13]. In all such children, revaccination after infancy is essential to improve the chances of seroconversion. The injectable vaccine currently used is not readily adaptable to house-to-house administration. Microneedle patch vaccine technologies currently under development might permit house-to-house visits during supplemental immunization activities (SIAs), with higher vaccination coverage the likely result [14].

### Issues specific to endemic countries

### Issues related to measles vaccination policy

Developed countries often set their own policies based on review of national data and, where available, national research. Most give measles as part of a combination vaccine, such as measles-mumps-rubella vaccine, MMR. On the other hand, developing countries generally adhere to the W.H.O. technical guidelines, sometimes with modifications. For decades, the WHO Expanded Programme on Immunization (EPI), recommended a schedule based on a single dose of measles vaccine, usually given at 9 months of age. This was a recommendation for control and for mortality reduction. It worked well wherever infant completion rates were high. However, infant dropout rates have remained a persistent problem in many countries. Moreover, health education has, until recently, emphasized the need to complete all vaccinations in the first year of life, advice no longer applicable in countries where the second dose of measles containing vaccine (MCV2) is now administered at 15 or 18 months of age. The most recent effort to overcome the dropout problem has been the use of SMS reminders to mothers and other caregivers, already implemented on a pilot basis in such countries as Kenya, Nigeria, and Zimbabwe [15-17].

In addition to efforts at raising routine infant vaccination coverage, the current century has seen two policy changes: 1) Introduction of the “second opportunity” to vaccinate against measles, interpreted by most countries as periodic (usually triennial) campaigns to vaccinate susceptibles regardless of their vaccination history. This takes place through mass vaccination of, typically, 9 to 59-month-olds. Such campaigns serve the purpose of vaccinating the previously missed, and of conferring immunity on the minority of non sero-converters among those already vaccinated. 2) The second year of life (2YL) approach to vaccination, with a second routine dose of measles containing vaccine given in the second year of life. The 2YL approach reduces the risk of outbreaks in the two to three years between campaigns among under-vaccinated children. It aims at elimination of the 15 percent children vaccinated at 9 months of age, who do not seroconvert.

The 2YL approach works only in those countries where the caregivers, oriented for many decades to bring in their children during infancy, are fully aware of the need to bring in the already vaccinated child for revaccination at 15 or 18 months of age. This means more efforts at communication than have thus far been done by many programmes in developing countries. Although, infant vaccination programmes can fall short in 2YL measles coverage, it has been demonstrated in several African countries that improvements in MCV2 occur over time.

## SIA implementation issues

Most international partners are aware that eradication requires both good routine immunization and high quality Supplemental Immunization Activities (SIAs). Notably, SIAs have been a major component of the polio eradication strategy and have, in the current century, almost always been done house to house, frequently using laypersons to administer the oral polio vaccine. The model for measles SIAs as part of the measles elimination strategy originated with the Pan American Health Organization, which in the 1990s developed the combination of catch-up SIAs, typically aimed at under-15-year-olds, periodic follow-up SIAs, case-based surveillance, and effective case management [18]. The PAHO model, adapted, worked well in southern Africa, starting in 1996 [19], though recent years have seen setbacks.

Measles SIAs have followed the lead of polio SIAs in some but not all respects. Because of operational problems with the house-to-house use of injectable vaccines, most measles SIAs have been done from existing health facilities, sometimes using temporary fixed vaccination posts set up, in markets, schools and places of worship. House to house social mobilization in support of measles SIAs began in the Region of the Americas. It has since spread to Africa and Asia, with support from the American Red Cross and the International Federation of the Red Cross/Crescent.

High coverage is needed for SIAs to achieve the interruption of measles transmission. To achieve high coverage, W.H.O. has developed planning and preparedness guidelines. The practical impact of the preparedness guidelines has been to force governments to quantify, by a scoring system, whether they are ready to keep their scheduled SIA dates or, when preparations are late, to reschedule. Kenya’s preparedness exercise before the 2016 SIA led the government to reschedule the SIAs, which then achieved 95 percent coverage. The measles SIAs implementation guidelines, in line with the Global Measles/Rubella Elimination Plan (2012-2020), emphasizes integrated approaches to achieve and maintain very high levels of population immunity using both routine immunization and SIAs, a significant difference with the initial drives towards Polio that were focused on largely using SIAs. Where religious or ethno-linguistic minorities object to vaccinations, efforts are necessary to quantify vaccination coverage among them and, as needed, take corrective measures [20,21].

## Management issues at the national level

Measles endemic countries have greatly varying health systems, and, at the country level, important intra-country differences. Some, such as Somalia, have, historically, required heavy external inputs to achieve elimination. In that country, financial support for smallpox eradication and polio elimination came almost entirely from international agencies. High and middle income countries, such as South Africa, have required little outside support to achieve disease elimination and eradication. In between, lies countries like India with most financing from in-country, and technical support from W.H.O. and other partners.

As developing countries assume more of the financing responsibility for their vaccination programs, vaccine advocates need to point out the economic benefits of vaccination against measles and other vaccine preventable diseases. One recent analysis places the return on investment for measles vaccination at $58 in benefits per dollar of investment [22].

Recent decentralization of SIAs planning and management can pose problems when some provinces or regions are firmly committed to eradication, while others lack either the resources or the political commitment to finish the job. In both smallpox and polio eradication, the states of southern India were the first to stop disease transmission, while the poor and populous northern states, especially Bihar and Uttar Pradesh, presented repeated challenges to the eradicators. In Nigeria, the southern states, with more resources and higher levels of maternal education, were the first to stop polio transmission. The virus persisted in the northern states, with low level transmission going undetected in Borno State as late as 2016. In the case of measles, no Nigerian state has yet interrupted measles transmission, but most southern states have generally lower measles incidence rates, primarily because of higher levels of routine immunization. Ethiopia, with large nomadic areas, especially in Somali region, presents low levels of routine immunization in pastoral areas, and relatively higher coverage in urban and agrarian areas.

In all countries, the task of measles eradication is to level the playing field, so that places such as Bihar in India and Somali Region of Ethiopia will have coverage approaching 100 percent at the time of global eradication. Unlike in polio and smallpox eradication programs, equitable routine and SIAs vaccination coverage would be a requirement to measles eradication

The problem of vaccine hesitancy, especially in industrialized countries, has come to the fore when diseases once common have become so rare that vaccination no longer commands the instant attention that it did when, amid conditions of high endemicity, the vaccines were first introduced. This phenomenon, not yet a major obstacle to measles vaccination in most Asian and African countries, continues to pose a major impediment to polio eradication in all three of the remaining endemic countries (Nigeria, Afghanistan and Pakistan). And notably, would make measles eradication more difficult after prolonged periods of measles elimination.

### Issues specific to international partners

Of great interest to international partners are the comparative costs of eradication programs requiring time limited investments versus long term control. There are no global estimates either of the costs of time limited measles (or measles/rubella) eradication. Thompson and Odahowski estimated that routine immunization and supplemental immunization activities will cost governments and donors $US 2.3 billion per year to vaccinate global birth cohorts of approximately 134 million surviving infants [23]. Such findings on the costs of long term control in the industrialized countries need emphasis. Some western countries lack enthusiasm, both for measles elimination within their own borders and measles eradication on a global scale. Yet these countries are among those with the most to gain by global eradication requiring a time limited effort. A multi-country evaluation of measles eradication and an evaluation from Uganda documented that Measles eradication by 2020 would be the most cost-effective scenario, both in the six countries and globally. Notably, eradicating measles by 2020 was projected to cost an additional discounted $7.8 billion and avert a discounted 346 million DALYs between 2010 and 2050 [24]. A similar study on measles elimination in Uganda by 2020 was projected to avert 130,232 measles cases, 3520 measles deaths, and 106,330 DALYs through the year 2030, compared with the next best scenario (95% mortality reduction by 2015), and was judged as the most cost-effective strategy [25].

### Organizational issues

The Measles and Rubella Initiative (MRI) and its forerunner, the Measles Partnership have, since 2001, served as a coordinating mechanism for the five original partners (American Red Cross, Centers for Disease Control and Prevention (CDC), UNICEF, the UN Foundation, and W.H.O.) and a growing number of newer MRI partners. MRI now counts >20 partners, and has collaborated in reducing the reported annual measles mortality from 853,479 in 2000 to 254,928 in 2015 [26]. In polio eradication, as in smallpox, no such a coalition of international partners existed before a WHA resolution. Whether global eradication will require a modification to the existing organizational framework remains to be seen.

Organizational culture has evolved almost beyond recognition since the first global eradication efforts of the 1950s. In his discussion of previous eradication efforts, D. A. Henderson pointed to the top-down organization of the malaria programs of the ‘50s and ‘60s. The program was conceived and executed as a military operation to be conducted in an identical manner whatever the battlefield. Involvement of the community or of persons at the community level was not part of the overall strategy [27].

Times have since changed, and most vaccination programs are run on a less rigid and more participatory basis than the first malaria programs. Community participation, especially the use of volunteers to sensitize the public, is a current feature in many programmes, and likely to be more pronounced in future global efforts. One salient feature of the Global Polio Eradication Initiative, GPEI, has been the CDC deployment of STOP (Stop Transmission of Polio) teams to countries needing additional technical support, especially for surveillance and communication. The STOP model, useful to GPEI, is been adapted to measles elimination and will more likely be for eradication.

## The role of innovations in eradication

Both smallpox and polio eradication benefited from innovations; one thinks of the introduction of surveillance and containment in the Smallpox Eradication Programme (SEP) and the switch from trivalent to bivalent OPV in the Global Polio Eradication Initiative (GPEI), neither envisaged when the respective programs were launched. If smallpox and polio are any indication, the launching of a global measles eradication initiative will stimulate innovation. Thus, for example, the introduction of bifurcated needles in SEP, and the introduction of bOPV in GPEI were responses to the needs of global eradication.

A review of past and possible future innovations in measles eradication could be: Preparedness exercises before SIAs, with rescheduling when benchmarks are not met; Wide age range SIAs [28]; Use of W.H.O. guidelines, especially “Planning and Implementing High Quality Supplemental Immunization Activities for Injectable Vaccines using the Example of Measles and Rubella Vaccines” [29]; House-to-house messaging during SIAs by volunteer announcers/community mobilizers [29]; Use of mapping with GIS techniques for SIAs; Use of auto-disabled syringes for vaccine reconstitution and for vaccination; Whole genome sequencing and nomenclature of measles and rubella viruses [30]; Rapid diagnostic tests; Eradication investment case as an advocacy and planning tool [31]; Mobile phone technology [32]; Use of measles risk assessment tools [33]; Containment of transmission through targeted vaccination [34]; Use of patch vaccinations for routine and SIA immunization [35]; Use of SMS reminders in routine immunization [36]; Use of SMS alerts during SIAs; Application of STOP team methods to measles; Revisions to International Health Regulations (IHR).

### Issues based on comparison with previous eradication programs

As of this writing, the world is approaching the end of the third decade of what was to have been a 12 year polio eradication effort. The Global Polio Eradication Initiative, GPEI, has done far worse than its predecessor, smallpox eradication, with missed deadlines. The missed targets associated with polio eradication have resulted in cost overruns that have discouraged donor and partner agencies from fully embracing the call for global measles eradication. The differences between ME and previous eradication programs were addressed during the 2010 feasibility consultation. Notably, measles eradication programme would be easier than some prior eradication programs, based on some technical and social factors and due to the progress towards elimination already made in the Americas and other regions. Measles eradication would also be cheaper than polio eradication and the failed malaria eradication efforts [37].

The following table summarizes the similarities and differences among measles and two other eradicable diseases [38]. In terms of biological and technical feasibility, measles is closer to smallpox than to polio. Surveillance is relatively easy because the case to infection ratio is close to 1:1. Environmental sampling is not necessary, since there is no excretion of vaccine virus. There is no vaccine derived virus. In these respects, measles is an easier target than polio. The basic reproductive number, 9 to 18, makes measles a challenging eradication target. The comparable figures for polio and smallpox are 4-13 and 5-7 respectively [39]. The expected external financing requirement for a time-limited measles eradication program is also estimated to be US$7.8 billion [40] which is 5-6 times more than the estimated cost of a 12-years Polio Eradication program Table 2.

**Table 2:** Differences between Smallpox, polio and measles eradication programs

	Smallpox	Polio	Measles
**Biological and technical feasibility**			
Etiologic agent	Virus	Virus	Virus
Nonhuman reservoir	No	No	No
Effective intervention tool	Smallpox vaccine	Oral vaccine	Injectable vaccine
Simple/practical diagnostic	Clinical diagnosis, confirmed by microscopy (If needed)	Stool culture	IgM
Sensitive surveillance	Facility and community based	Facility-based	Facility based
Field-proven strategies	Americas, West Africa	Americas	Americas
**Cost and Benefits**			
Cases averted per year	>100,000	350,000	>100,000,000
Coincident benefits	Creation of Expanded Programme on Immunization	Improved immunization and bio-surveillance	Improved routine immunization and surveillance
Intangible benefits	Culture of prevention and social equity	Culture of prevention and social equity	Culture of prevention and social equity
Estimated annual direct global savings	>$100 million per year; averted	US$1.5 billion	>US$2 billion
Estimated total external financing	c. $100-125 million	US$2.0-2.5 billion (as of 2000)	$7.8 billion [39]
**Social and political considerations**			
Political commitment (endemic/industrial countries)	Variable/strong	Variable/strong	Variable
Social support (endemic/industrial countries)	Variable/strong	Variable/strong	Variable
Core partnerships and advocates	WHO, CDC	WHO, Rotary, CDC, UNICEF	WHO, CDC, UNICEF, American Red Cross, UN Foundation
Technical consensus	WHA resolutions	World Health Assembly	Regional resolutions

Note: The first and third columns are reproduced from R. B. Aylward et al., “When is a Disease Eradicable? 100 Years of Lessons Learned”. The second and fourth columns have been added for this article.

The most notable differences between polio and measles are in the third grouping of criteria, “social and political considerations.” Political commitment to measles eradication is strong in the Region of the Americas and variable in the five other regions of W.H.O. It is in the interests of an accelerated eradication effort to disseminate more widely information on the permanent economic benefits of eradication, both in developed and developing countries. Donor contributions should be regarded as investments, and not merely as expenditure.

## Justification for a short, time-limited eradication effort

Among points raised at the Washington consultation of 2010 was the duration of the measles eradication effort. One question, raised by Cutts and Steinglass, among others, relates to the age range of susceptibles, most of whom need to be vaccinated before transmission can be interrupted [41]. This, in turn, relates to the duration of the eradication effort. An overextended eradication effort could push the age of susceptibles into young adulthood, with unfortunate consequences. In the end stages of global measles eradication, would it be necessary to immunize every susceptible, as has been done with Polio or only to identify transmission chains and conduct focal immunizations? If the latter, then an adaptation of smallpox containment strategies fits measles well.

If a global eradication effort took much more than 5-10 years, the likely accumulation of susceptibles in adolescents and adult age groups might become challenging, especially in countries with anti-vaccination movements. All of these considerations could lead either to hesitation on the part of countries before voting a W.H.A. resolution, or to inadequate external funding for national efforts, once a W.H.A. resolution was passed. Examples are not lacking of member states voting for eradication, then pulling back from their original commitments.

One way to strengthen organizational and government commitments will be the development of a formal Eradication Investment Case (EIC), proposed by Thompson and colleagues [42] and currently under development. Development of an investment case should be completed prior to launch of the eradication initiative. The EIC should support and inform deliberations and decisions made by national health leaders at the World Health Assembly and elsewhere, as they consider a global commitment to an eradication goal. An EIC will also stimulate the development of an eradication financing plan, as stakeholders evaluate the choice to commit or not to an eradication goal.

If measles were eradicated after polio, it would be (with or without rubella, depending on the global target), the third viral disease to be globally eradicated [43-45]. Measles and rubella could be the third and fourth human diseases to be eradicated. Although political commitment to rubella eradication is far from universal, two factors militate in favor of a joint measles/rubella eradication initiative: 1) The relative simplicity of switching antigens (already done in many developing countries) while retaining the same mix of routine and SIA strategies as with measles; 2) The use of the same or nearly identical surveillance and laboratory mechanisms for rubella and measles diagnostics as for measles alone. Writing in 1998, Strebel listed the following challenges to global measles eradication: “perception in developed countries that measles is a minor disease of little consequence; lack of political and financial support; ease of importation of measles virus, particularly through air travel, and need to mobilize global resources and collaboration among partner organizations and focus these over a relatively short period of time (3-5 years)” [45]. Two decades later, that list of challenges remains essentially the same.

The history of previous global eradication efforts is a history of missed deadlines. The WHA malaria resolution of 1955 is still unfulfilled as of today. The 1959 smallpox resolution by the World Health Assembly saw continuing transmission at the country level until 1977. The polio eradication effort, targeted for completion in 12 years, is now in its twenty-ninth year. With measles, because of the disease’s epidemiology, it should be different. If previous eradication efforts have been marathons, measles eradication will need to be a sprint.

## Competing interests

The authors declare no competing interest.
